# Supplementary material for: Getting More Out of Biomedical Documents with GATE's Full Lifecycle Open Source Text Analytics
Source: PLoS Comput Biol. 2013 Feb 7;9(2):e1002854. doi: 10.1371/journal.pcbi.1002854 (PMC3567135; doi:10.1371/journal.pcbi.1002854)
Supplement: Dataset S1 — GATE software. Dataset S1 bundled with this paper contains a distribution of GATE (or see http://gate.ac.uk/download/). (TGZ) [file pcbi.1002854.s001.tgz › plos-gate/src/gate/resources/texts/news/telecoms-gua-16-May-2001.html]

Guardian | Grabiner leaves top job at Energis

**Grabiner leaves top job at Energis**


Chief executive rules himself out as successor to BT's Boneld and vows to leave telecoms sector

**David Teather, media business editor**  
**Wednesday May 16, 2001**  
**The Guardian**

Energis chief executive Mike Grabiner stunned investors yesterday when he announced his departure from the firm he has built from an underperforming part of the National Grid to a fixture in the FTSE 100.

Mr Grabiner, who will step down in July, immediately ruled himself out as a potential successor to BT's beleaguered chief executive, Sir Peter Bonfield. The highly regarded executive had been widely tipped as a possible candidate should Sir Peter leave BT before the end of his contract in December 2002.

Mr Grabiner, who cashed in share options worth nearly £9m in November 1999, said he would be taking some time off to consider what his next step should be.

"I have always felt that if I could build Energis to proven success, I would consider doing something totally different with the rest of my career," he said. "I have no job to go to and no idea what I'll end up doing. I just know I'm 50 and I want to do something different while I'm young and energetic enough to do it. The BT job is not for me. I've spent 27 years in telecoms and I don't want to run another telecoms company."

Mr Grabiner will receive no pay-off but before he leaves will cash in around £3.2m from exercising options on 1.6m shares awarded to him last year. He still has around 440,000 shares.

Mr Grabiner's departure coincided with Energis moving into pre-tax profit for the first time in its domestic market. The company made £3.2m in the UK for the year to April although younger operations in mainland Europe dragged Energis into a pretax loss of more than £100m. The company is forecasting a pretax profit from its overseas operations in the 2003-2004 financial year.

Turnover of £840.4m was 70% higher compared with the previous year.

Mr Grabiner joined Energis as chief executive in 1996 and guided the company from an underperforming division of the National Grid through to a successful stock market flotation in 1997. The company is worth £4.7bn.

When he took over, the business had revenues of just £42.7m and losses at the Ebitda (earnings before interest, taxes, depreciation and amortisation) level of £42.6m.

His only other job had been at BT. He joined the Post Office as a graduate trainee in its telecoms department before it was spun off as BT and rose to the job of director of its European operations.

Shares in Energis fell 9% in early trade. "It's not very good news at all for the company," said one analyst. "He has a fantastic reputation in the market." By the close, however, they had recovered their loses and were 5p higher at 276p.

Mr Grabiner will be replaced by chief operating officer David Wickham, who joined Energis from Cable & Wireless at the end of 1999. At C&W he had been in charge of delivering a single international voice and internet capability for the group.

Guardian Unlimited © Guardian Newspapers Limited 2001
